# The *Bombyx mori singed* Gene Is Involved in the High-Temperature Resistance of Silkworms

**DOI:** 10.3390/insects15040264

**Published:** 2024-04-12

**Authors:** Zhenye Liu, Cong Li, Wenyu Yang, Qiao Wu, Wenfu Xiao, Yan Zhu, Qiongqiong Wei, Zhanqi Dong, Guizheng Zhang, Cheng Lu, Minhui Pan, Peng Chen

**Affiliations:** 1 State Key Laboratory of Resource Insects, Southwest University, Chongqing 400715, China; m15138643580@163.com (Z.L.); 14799010068@163.com (C.L.); m15993968206_1@163.com (W.Y.); wuqiao575757@163.com (Q.W.); mangzishijia@126.com (W.X.); zhuyan0806@126.com (Y.Z.); qiongqiongwei@126.com (Q.W.); zqdong@swu.edu.cn (Z.D.); lucheng@swu.edu.cn (C.L.); 2 Sericultural Research Institute, Sichuan Academy of Agricultural Sciences, Nanchong 637000, China; 3 Guangxi Key Laboratory of Sericultural Genetic Improvement and Efficient Breeding, Sericulture Technology Promotion Station of Guangxi, Nanning 530007, China; zhangdoudou1999@163.com

**Keywords:** silkworm, high temperature stress, *singed* gene, oxidative stress, DNA damage

## Abstract

**Simple Summary:**

Temperature is an important factor that affects the growth and reproduction of insects. The silkworm is an economically important insect, and as a representative insect of Lepidoptera, it is particularly important to investigate its extreme-temperature resistance mechanisms. In this article, we identified a gene in *B. mori*, the *B. mori singed* (*Bmsn*) gene, which is involved in the high-temperature resistance of silkworms. We demonstrated that the *Bmsn* gene can increase the proliferation activity of silkworm cells and enhance their tolerance to high temperatures. Furthermore, we constructed a transgenic *B. mori* strain that overexpressed the *Bmsn* gene and demonstrated that its overexpression can increase high-temperature resistance and improve the economic characteristics of silkworms.

**Abstract:**

Temperature is an important factor in the growth, development, survival, and reproduction of organisms. The high-temperature resistance mechanism of insects may be significant for use in the prevention and control of insect pests. The silkworm, *Bombyx mori*, is an important Lepidoptera model species for studies on pest control in agriculture and forestry. We identified a gene in *B. mori*, the *B. mori singed* (*Bmsn*) gene, which is involved in the high-temperature resistance of silkworms. Sn proteins are highly conserved among species in many taxonomic groups. The overexpression of the *Bmsn* gene promoted the proliferation of silkworm cells, reduced oxidation, and reduced the accumulation of reactive oxygen species under stress. Interfering with the *Bmsn* gene had the opposite result. We constructed a transgenic *B. mori* strain that overexpressed the *Bmsn* gene. The physiological traits of the transgenic strain were significantly improved, and it had stronger high-temperature resistance. The *Bmsn* gene is involved in the process by which fat bodies respond to high-temperature stress. These findings provide insights into the mechanism of high-temperature resistance of insects and offer a new perspective on agricultural and forestry pest control.

## 1. Introduction

Almost all living things undergo adaptive changes in response to external stimuli to maintain their health and normal life activities. These stimuli include temperature changes, chemical stimuli, and microbial infections [1]. Insects are a diverse and widely distributed group. Temperature is a key factor that has a great impact on insect populations and distributions. Insects are ectothermic, and they rely on external heat sources to regulate their body temperature [2]. As a result, they are more sensitive to changes in ambient temperature than endothermic animals. The behavior, growth, and metabolism of insects are influenced by variations in ambient temperature [3]. For example, many insects adapt to changes in ambient temperature by regulating their respiration until they reach a critical temperature limit [4].

Heat stress is the most conserved adaptive response in organisms [5]. Heat stress under natural conditions usually has a short duration due to diurnal temperature variations. Under heat stress, insects tend to reduce their activity to lower their metabolism, thus ensuring their survival in high temperatures [6]. However, when faced with prolonged extreme heat, insects often suffer irreversible damage [7]. Heat stress leads to the accumulation of reactive oxygen species (ROS). The accumulation of ROS causes oxidative stress, which results in DNA damage, lipid oxidation, protein degradation, enzyme inactivation, and, ultimately, cell death [8]. Insects regulate their body metabolism via a heat stress response to adapt to high-temperature environments [9]. When the ambient temperature increases, a variety of regulated factors participate in the stress response, including heat shock proteins (HSPs) and activating transcription factor 2 [10,11].

The global climate is warming, and increased environmental temperatures pose a threat to the normal activities of many insects. High-temperature shock can alter the intestinal microbial composition and metabolism of insects, subsequently affecting their resistance, growth, development, and nutrient absorption [12,13]. For example, high temperatures can alter the balance of lactic acid bacteria in the intestinal tract of *Drosophila*, leading to a shorter lifespan [14]. The silkworm, *Bombyx mori*, is an important economic insect that produces silk [15]. High temperature is a significant factor contributing to the decline in sericulture production. More than half of all pests of agriculture and forests belong to Lepidoptera. The silkworm is a representative Lepidoptera species and a useful biological model for research on pest control [16]. High temperatures affect the absorption of nutrients and shorten the fifth instar growth period of silkworms [17]. In the silkworm, interfering with the *BmGrpE* (XM_004926291.4) gene of the Hsp transcription factor can enhance its high-temperature resistance [18]. However, the mechanism of high-temperature resistance of silkworms is incompletely known.

To study the mechanism of silkworm response to high temperatures and identify the key genes that contribute to high-temperature resistance, we previously identified 12 differentially expressed genes via transcriptome sequencing analysis of two silkworm strains with different high-temperature sensitivity [19]. The *B. mori singed* (*Bmsn*) gene, a member of the fascin family, was upregulated fourfold in resistant silkworm strains at 24 h of high-temperature treatment. These data suggested its involvement in the silkworm response to high temperatures. In the present study, we investigated the role of the *Bmsn* gene in the high-temperature resistance of silkworms. Our results showed that the overexpression of the *Bmsn* gene enhanced the proliferation activity of silkworm cells, decreased the accumulation of ROS caused by oxidative stress, repaired DNA damage in cells, and extended cell survival, whereas interfering with the *Bmsn* gene had the opposite results. At the individual level, a transgenic strain overexpressing the *Bmsn* gene (*Bmsn*-OE) was constructed, and our data showed that transgenic strains can significantly increase the survival rate at high temperatures and the cocoon layer quantity. We also revealed that fat bodies play an important role in the high-temperature response of *Bmsn*-OE strains. Our results provide a new perspective for understanding the heat tolerance mechanism of insects and new targets for the control of agricultural pests.

## 2. Materials and Methods

### 2.1. Gene Analysis and Vector Construction

The *Bmsn* gene sequence (XM_038017024.1) was obtained from the National Center for Biotechnology Information (NCBI, https://www.ncbi.nlm.nih.gov/, accessed on 2 December 2020) and SilkDB3.0 (https://silkdb.bioinfotoolkits.net/, accessed on 10 December 2020). The structure of the Bmsn protein was predicted using the SMART website (http://smart.embl-heidelberg.de/, accessed on 15 December 2020), and the homologous sequence of the Bmsn protein was compared using Clustalx1.83 and GenDoc2.7 software. A phylogenetic tree was constructed using Clustalx1.83 software and MEGA6.0 software, and it was visualized using iTOL v6 (https://itol.embl.de, accessed on 5 April 2021). The *Bmsn* gene with a Flag tag was cloned onto the eukaryotic expression vector pIZ/V5-His carrying green fluorescent protein (GFP), and transcription was driven by the OpIE2 promoter. The siRNA targeting the *Bmsn* gene for the interference vector was predicted using the BLOCK-IT interference RNAi Designer website (http://rnaidesigner.thermofisher.com, accessed on 20 September 2021). The gene was cloned into the pIZ/V5-His vector after synthesis. The primers used in this study are listed in Table S1.

### 2.2. Cell Culture and Transfection

The silkworm BmN-SWU1 cell line [20] was cultured at 27 °C in TC-100 insect cell culture medium (US Biological, Swampscott, MA, USA) supplemented with 10% fetal bovine serum (FBS; BIOAGRIO, S1356). Transfections were performed at ratios of 2 µg/6-well plates (4–8 × 10^5^ cells), 1 µg/12-well plates (1.6–3.2 × 10^5^ cells), and 0.5 µg/24-well plates (0.8–1.6 × 10^5^ cells). The ratio of the plasmid to transfection reagent (Roche, Shanghai, China) was 1 μg to 2 μL.

### 2.3. Treatment of Silkworms

The Dazao strain was provided by the Gene Bank of Domestic Silkworm Resources of Southwest University. In the experimental group exposed to the hyperthermic shock, the larvae were kept at 37 °C, while the control group was kept at 27 °C. Both groups were fed with fresh mulberry leaves. The experimental group (~0.8 g) was injected with a 20 μL solution of 3% H_2_O_2_ into the fourth abdominal spiracle. The control group was injected with 20 μL of PBS solution (0.01 M). The fat body (~8–11 mg) was collected at different time points, washed twice in phosphate buffer (0.01 M), placed in a 1.5 mL RNA-free centrifuge tube, and stored at −80 °C.

### 2.4. Total RNA Extraction

Total RNA was extracted from cells and tissues according to the Total RNA Kit (Omega Bio-Tek, Guangzhou, China). The tissues were ground with a sterilized mortar, and the cells were collected in a 1.5 mL RNA-free centrifuge tube. We then added an appropriate amount of lysate. The entire process was conducted on ice to prevent RNA degradation. After extraction, the RNA pellet was dissolved in 30–40 μL of RNA-free water and stored at −80 °C.

### 2.5. Quantitative Real-Time PCR (qRT-PCR)

The cDNA for qRT-PCR was synthesized from the previously prepared total RNA above using the PrimeScript™ RT Reagent Kit (Takara, Beijing, China). qRT-PCR was performed using Hieff^®^ qPCR SYBR Green Master Mix (Yeasen, Shanghai, China) and the CFX96™ Touch Real-Time PCR System (Bio-Rad, Berkeley, CA, USA). The reaction conditions were 95 °C for 30 s, 95 °C for 5 s, and 60 °C for 30 s, for a total of 40 cycles. Three biological replicates were performed for each sample. The internal reference gene was the Eukaryotic translation initiation factor 4A (SW22934) (Table S1).

### 2.6. Detection of ROS

We used the ROS kit (Beyotime, Shanghai, China) to detect the level of ROS in cells and tissues. DCFH-DA (2′,7′-dichlorodihydrofluorescein diacetate) was diluted to 10 μM with serum-free medium. We then added the appropriate amount of solution, according to the type of well plate, and incubated for 20–30 min at 37 °C, protected from light. The cells were observed with a laser confocal microscope after incubation. The cells were treated similarly; they were added to a 96-well enzyme labeling plate, and the fluorescence value was detected using a multifunctional microplate reader (BioTek, Winooski, VT, USA). A total of 20 µL of the above solution was taken to determine the protein concentration, and the relative ROS level was calculated as the fluorescence value/protein concentration. qRT-PCR was used to detect the expression of *BmSOD1*, *BmSOD2*, *BmSOD3*, *BmCAT*, *BmGpx*, and *BmGADD45* (Table S1).

### 2.7. Cell Proliferation Activity Detection

We used a Cell Counting Kit-8 kit (CCK-8) (Beyotime) to measure cell viability according to the manufacturer’s instructions. First, walled cells were gently suspended by pipetting 100 µL (2000 cells) into a 96-well plate. Subsequently, 10 µL of CCK-8 reagent was added to each well in complete darkness. Each group of samples was repeated three times. The samples were then incubated in an incubator at 37 °C for 4 h, after which the absorbance was measured at 450 nm using a Multifunctional enzyme marker (BioTek, Winooski, VT, USA).

### 2.8. Single Cell Gel Electrophoresis Assay

We used the DNA Damage Comet Assay Kit (Keygentec, Nanjing, China) to detect whether the accumulation of ROS caused by hyperthermic treatment of BmN-SWU1 cells for 12 h would cause DNA damage and, if so, the degree of damage. The DNA-damaged cells were analyzed in detail by Casplab2.0 software. A total of 30 cells in each group were selected to measure the length of the comet tail, the ratio of DNA content in the head and tail to total DNA content, and the distance from the tail to OTM (the distance between the tail and cell).

### 2.9. CalCEIIN-AM/PI Fluorescent Double Staining

The survival of BmN-SWU1 cells treated with a high temperature was detected by a Calcein-AM/PI living cell/dead cell double staining kit (Yeasen). The cells were treated according to the kit instructions. A 490 ± 10 nm amount of excitation filter was used to detect both living cells (yellow-green fluorescence) and dead cells (red fluorescence) under a fluorescence microscope.

### 2.10. Construction of Transgenic Vector of Silkworm

The *Bmsn* gene with a Flag tag was constructed on the vector piggyBac [3 × P3 EGFP], along with green fluorescent protein. This vector was mixed with the helper plasmid (pHA3PIG) at a molar ratio of 1:1 and microinjected into silkworm eggs to obtain the *Bmsn* overexpression transgenic strain. Then, by detecting the insertion site, the genes on the left and right sides of the insertion site were determined and their expression levels were detected by qRT-PCR.

### 2.11. Statistical Analysis

All experiments were performed at least three times. All data were expressed as the mean ± standard deviation (SD) of three independent experiments. All of the statistically significant differences between treatments were determined using Student’s *t*-test. A value of *p* < 0.05 (*) was considered statistically significant, and *p* < 0.01 (**) was considered very significant.

## 3. Results

### 3.1. Identification and Characteristic Analysis of the Bmsn Gene

**Figure 1:**
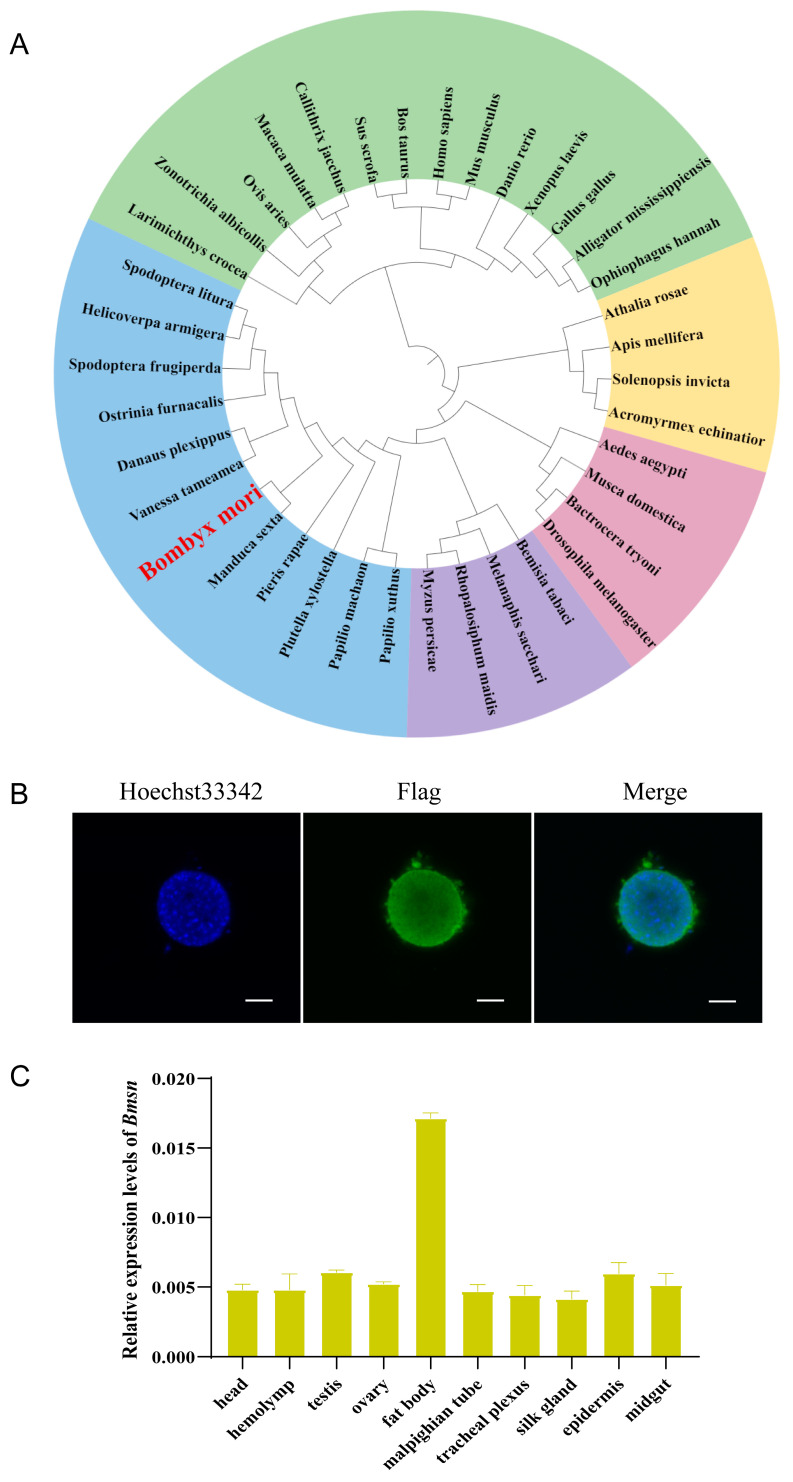
Identification and characteristic analysis of the *Bmsn* gene. (**A**) Phylogenetic analysis of *Bmsn*. Green indicates vertebrates, pink indicates Diptera, blue indicates Lepidoptera, purple indicates Hemiptera, and yellow indicates Hymenoptera. (**B**) Fluorescence subcellular localization of Bmsn protein in BmN-SWU1 cells, Scale bar = 5 µm. (**C**) Quantitative analysis of *Bmsn* gene expression in different tissues of silkworm larvae on the third day of the fifth instar. The internal reference gene was the *Eukaryotic translation initiation factor 4A* (SW22934).

To identify the *sn* gene in the silkworm, we cloned this gene and found that it coded a protein of 511 amino acids containing four Fascin protein domains (Figure S1A). Multiple sequence alignment analysis showed that the sequences of sn were highly conserved among *Spodoptera litura*, *Helicoverpa armigera*, *Tribolium castaneum*, *Drosophila melanogaster*, *Apis mellifera*, *Mus musculus*, and *Homo sapiens* (Figure S1B). We examined the phylogenetic relationship of sn proteins by performing a phylogenetic analysis. Sn in vertebrates clustered on one branch, while insect species in the Lepidoptera, Diptera, Hymenoptera, and Homoptera clustered on individual branches. The sn of silkworm is most closely related to the sn of *Manduca sexta* (Figure 1A).

To analyze the expression characteristics of the *Bmsn* gene, we constructed a *Bmsn* overexpression vector and transfected it into BmN-SWU1 cells. Immunofluorescence results showed that the Bmsn protein was localized in the nucleus (Figure 1B). The expression levels of *Bmsn* were examined at the transcription level in tissues of the silkworm via quantitative and semi-quantitative PCR. Analysis of the expression pattern of the *Bmsn* gene in the tissues of silkworm larvae showed that the *Bmsn* gene was most highly expressed in the fat body (Figure 1C and Figure S2).

### 3.2. Effect of Bmsn on the Proliferation Activity of Silkworm Cells

**Figure 2:**
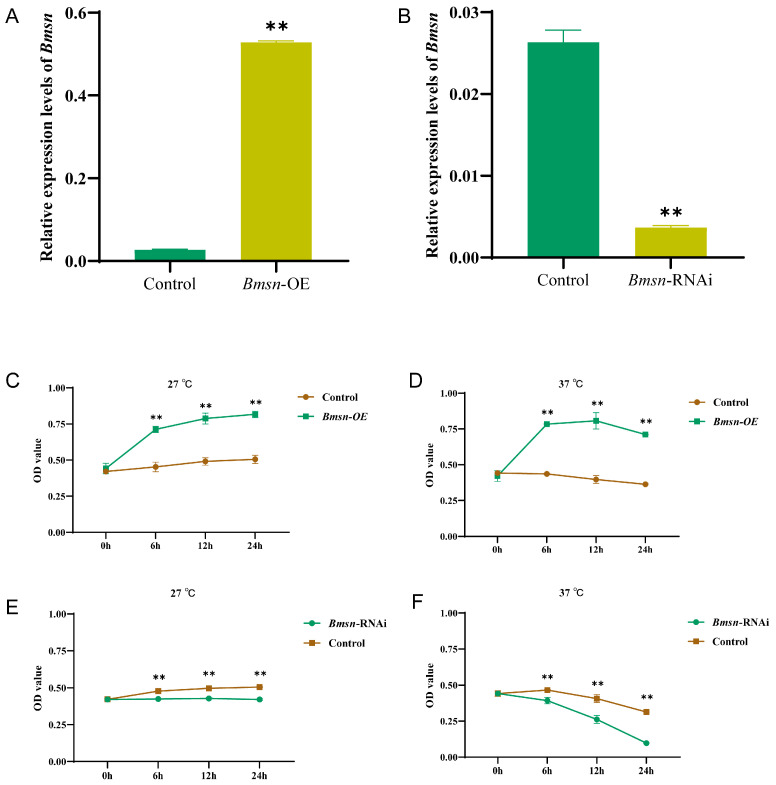
Effects of the *Bmsn* gene on cell proliferation. (**A**) Relative expression levels of the *Bmsn* gene after overexpression in BmN-SWU1 cells. (**B**) Relative expression levels of the *Bmsn* gene after interference in BmN-SWU1 cells. (**C**) Cell proliferation activities after overexpression of *Bmsn* gene at 27 °C. (**D**) Cell proliferation activities after overexpression of the *Bmsn* gene at 37 °C. (**E**) Cell proliferation activities after interference with the *Bmsn* gene at 27 °C. (**F**) Cell proliferation activities after interference with the *Bmsn* gene at 37 °C. **, *p* < 0.01, Student’s *t*-test.

We analyzed the effects of the *Bmsn* gene on the activity and proliferative capacity of silkworm cells after high-temperature treatment. The overexpression and interference vectors of the *Bmsn* gene were constructed and transfected into silkworm BmN-SWU1 cells. The expression levels of the *Bmsn* gene increased significantly after overexpression and decreased significantly after interference (Figure 2A,B).

Changes in cell activity and proliferative capacity occurred after overexpressing and interfering with the *Bmsn* gene in the cells. The proliferation of cells increased significantly with time at 27 °C after overexpressing the *Bmsn* gene compared to the control group (Figure 2C). After 37 °C treatment, the proliferation of cells increased significantly from 0 to 12 h compared to the control group. The proliferation activity was reduced after 12 h, but it was still significantly higher than that of the control group (Figure 2D). After interfering with *Bmsn*, cell proliferation decreased in all cases. Compared to 27 °C, the decrease in cell proliferation was more obvious after 37 °C treatment. These results indicate that interfering with the *Bmsn* gene significantly inhibited cell proliferation, while overexpression of the *Bmsn* gene significantly promoted cell proliferation, especially at a high temperature (Figure 2E,F).

### 3.3. Effects of the Bmsn Gene on ROS Levels and Cell Survival in BmN Cells

**Figure 3:**
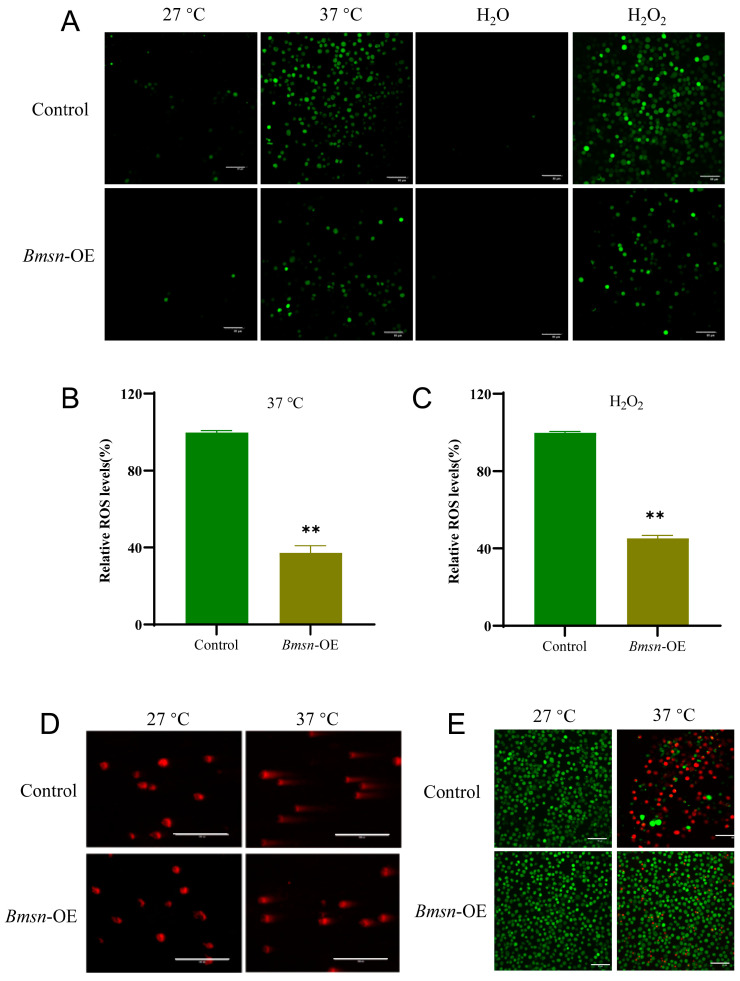
Effects of the *Bmsn* gene on ROS accumulation and cell survival in BmN-SWU1 cells. (**A**) After overexpression of the *Bmsn* gene, the ROS content was detected at a high temperature or after H_2_O_2_ treatment for 12 h. Scale bar = 80 µm. (**B**) The relative ROS content in the cells treated at 37 °C for 12 h was detected with an enzyme marker. (**C**) Relative ROS content in cells treated with H_2_O_2_ for 12 h was detected by an enzyme marker; **, *p* < 0.01, Student’s *t*-test. (**D**) Effects of the *Bmsn* gene on DNA damage in BmN-SWU1 cells under high-temperature stress. Scale bar = 40 µm. (**E**) Effects of the *Bmsn* gene on the survival rate of BmN-SWU1 cells under high-temperature stress. Scale bar = 80 µm.

To explore the effect of the *Bmsn* gene on oxidative stress, the *Bmsn* gene was overexpressed in silkworm cells. The level of ROS was then detected using a reactive oxygen fluorescence probe after the cells were treated with a high temperature or H_2_O_2_ for 12 h. The green fluorescence was significantly reduced compared to the control group after overexpression of *Bmsn*, indicating that the cellular ROS level was significantly reduced. The results measured by the enzyme marker were consistent with those observed by fluorescence microscopy (Figure 3A–C).

ROS accumulation can damage cellular nucleic acids, proteins, and lipids [21]. We determined whether the ROS accumulation caused by the high-temperature treatment of BmN cells after 12 h caused cellular DNA damage and the extent of the damage using a comet electrophoresis kit. The cells showed an obvious “comet-shaped tail”, as compared to the control group, after high-temperature treatment for 12 h, indicating that the cellular DNA was damaged (Figure 3D, Table 1). Overexpression of the *Bmsn* gene significantly reduced this damage. To clarify the ability of the *Bmsn* gene to reduce the damage caused by cellular oxidative stress, cell survival was examined after high-temperature treatment for 12 h. The living cells appeared green, and the dead cells were red under the fluorescence microscope. These results showed that the survival rate of cells after overexpression of *Bmsn* was significantly higher than the survival of the control group. After 37 °C treatment, a large number of cells in the control group died, while few cells died after overexpression of *Bmsn* (Figure 3E). This indicates that *Bmsn* can improve the survival rate of cells after oxidative stress.

### 3.4. Construction and Characteristic Analysis of Transgenic Silkworms Overexpressing the Bmsn Gene

**Figure 4:**
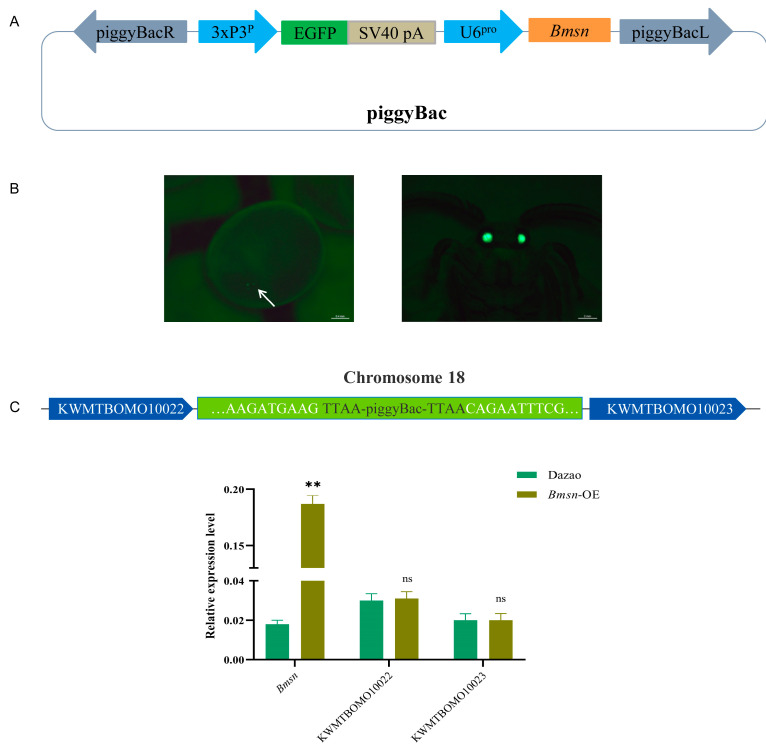
Establishment and analysis of insertion sites of the *Bmsn*-OE transgenic strain. (**A**) Schematic diagram of the *Bmsn*-OE transgenic strain vector construction. (**B**) The *Bmsn*-OE transgenic strain was screened positive, and the arrow indicates the green fluorescence in the eyes of the positive individuals, Scale bar (left) = 0.4 mm; Scale bar (right) = 2 mm. (**C**) Detection of the insertion site of the *Bmsn*-OE transgenic strain vector and expression analysis of the *Bmsn* gene and genes on both sides of the insertion site. **, *p* < 0.01; ns, *p* > 0.05, Student’s *t*-test.

**Figure 5:**
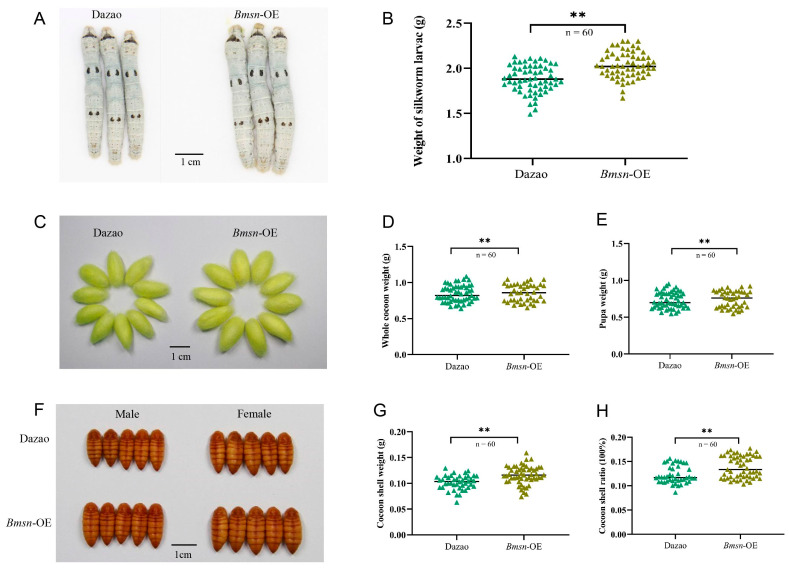
Analysis of the characteristics of *Bmsn*-OE transgenic strains. (**A**) Size of silkworm larvae on the sixth day of the fifth instar. Scale bar = 1 cm. (**B**) Body weight analysis of silkworm larvae on the sixth day of the fifth instar. (**C**) Cocoon size. Scale bar = 1 cm. (**D**) Statistical analysis of cocoon weight. (**E**) Statistical analysis of pupal weight. (**F**) Pupal size observation. Scale bar = 1 cm. (**G**) Statistical analysis of cocoon shell weight. (**H**) Statistical analysis of cocoon shell rate. **, *p* < 0.01, Student’s *t*-test.

To further explore the effect of the *Bmsn* gene on silkworm individuals, we constructed an overexpressing *Bmsn* transgenic silkworm strain, identified its reliability, and then analyzed the main traits of these transgenic silkworms. We successfully obtained a transgenic strain with overexpression of the *Bmsn* gene and demonstrated that the insertion of the *Bmsn* gene does not affect the expression of the adjacent genes *KWMTBOMO10022* and *KWMTBOMO10022* (Figure 4). Compared to the same developmental stage of the control strain (Dazao), the larval sizes and weights of the *Bmsn*-OE strain were higher than those of the control group (Figure 5A,B). This indicated that the *Bmsn* gene can promote silkworm growth. Sixty individuals from the *Bmsn*-OE strain and the Dazao strain were randomly selected, and the full cocoon weight, pupal weight, and cocoon layer weight were scored. The cocoons and pupae of the *Bmsn*-OE strain were larger than those of the control strain. The whole cocoon weight, pupa weight, cocoon layer weight, and cocoon layer rate were greater than those of the control strain. This indicated that the *Bmsn*-OE strain had improved economic traits (Figure 5C–H).

### 3.5. Effects of High Temperature on Transgenic Silkworms Overexpressing Bmsn Gene

**Figure 6:**
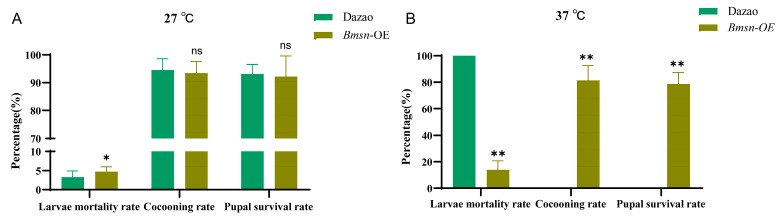
Tolerance differences between the *Bmsn*-OE strain and the Dazao strain. (**A**) Growth of *Bmsn*-OE and Dazao strains at 27 °C. (**B**) Growth of *Bmsn*-OE and Dazao strains at 37 °C. *, *p* < 0.05; **, *p* < 0.01; ns, *p* > 0.05, Student’s *t*-test.

We verified that the *Bmsn*-OE strain has improved economic traits. To further explore the high temperature tolerance of the *Bmsn*-OE strain, high-temperature shock experiments lasting three days were conducted on the two silkworm strains at the fifth instar. Analysis of the health index data of the *Bmsn*-OE strain and Dazao control showed that the average mortality in the *Bmsn*-OE strain was significantly lower than that of the control group. Under high temperatures, the cocooning rate and pupa life rate of the *Bmsn*-OE strain were significantly higher than those of the control group. There was no significant difference between the two strains at the normal feeding temperature (27 °C) (Figure 6). These results show that the high-temperature environment had a significant impact on the growth and development of the silkworm. The *Bmsn*-OE strain was less affected, indicating that overexpression of the *Bmsn* gene can help silkworms resist high temperatures.

### 3.6. Effects of Overexpression of the Bmsn Gene on the Silkworm Fat Body

**Figure 7:**
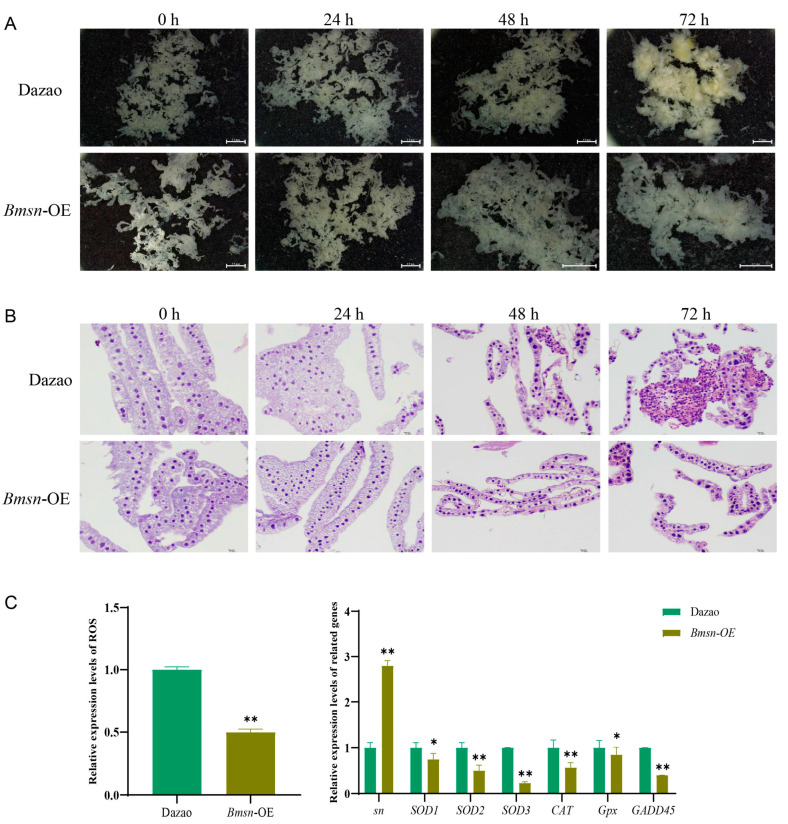
Effects of overexpression of the *Bmsn* gene on silkworm fat body. (**A**) Morphological changes in silkworm fat body after overexpression of the *Bmsn* gene. Scale bar = 2.5 mm. (**B**) Fat body HE staining. Scale bar = 10 µm. (**C**) Expression characteristics of fat body oxidative stress indexes and related genes in different strains. *, *p* < 0.05; **, *p* < 0.01, Student’s *t*-test.

High expression of the *Bmsn* gene occurs in the silkworm fat body (Figure 1C), so we investigated the detailed relationship between the *Bmsn* gene and the fat body. The morphological changes in the fat body of the experimental group (*Bmsn*-OE strain) and the control (Dazao) group were observed at different time points after high-temperature treatment. After 37 °C treatment for 48 h, differences between the fat body morphology of the control and the experimental group were apparent. After 72 h of 37 °C treatment, the fat body morphology of the control group was flocculent, while the fat body of the experimental group retained its flake-like shape (Figure 7A). This suggested that the *Bmsn* gene may play a role in delaying the degradation of the fat body. Changes in the fat body cells after high-temperature shock were also observed by HE staining. The fat body cells in the control group and the experimental group were densely arranged after 48 h of high-temperature treatment. The boundary between the adipose body cells in the control group then began to disappear, and the adipose body cells were dissociated after 72 h of treatment, while the cells in the experimental group were still densely distributed (Figure 7B). This suggests that overexpression of the *Bmsn* gene may delay the dissociation of fat body cells under high temperatures.

Changes in related indexes and related genes such as ROS, total SOD, and CAT were also detected in the fat body [22]. The oxidative stress index, ROS, total SOD, and CAT-related genes of silkworms treated with high temperatures for 24 h were significantly lower than those of the control group (Figure 7C). These data suggest that the *Bmsn* gene has a high-temperature resistance function by delaying damage to fat bodies caused by high-temperature stress.

## 4. Discussion

Insects are poikilothermic and generally have high-temperature requirements for their growth and development [23]. Under suitable temperature conditions, growth and development will accelerate with a temperature increase. However, when the temperature exceeds the suitable range, insect development will be retarded [24]. The reproduction of insects also requires a suitable temperature. Beyond this range, reproduction and reproductive capacity are inhibited [25]. High temperatures can cause changes in the structure and function of biological macromolecules and proteins in insects. It will affect their efficiency and function, including nuclear RNA transcription, translation, and RNA binding to ribosomal proteins [14,26]. Therefore, understanding the internal mechanism of high-temperature effects on insects is valuable for economically important insects and has significance for pest control. The silkworm is a useful biological model for research on pest control [27]. In this study, we demonstrated that the *Bmsn* gene is involved in the high-temperature resistance of the silkworm, and this provides a new target for further molecular improvement.

The *sn* gene encodes a single protein product that has homology with the myobundle protein (*Fascin*) from sea urchins [28]. The human *sn* gene is involved in cell proliferation [29]. Multiple sequence alignment analysis showed that the sequences of *sn* are highly conserved among different species (Figure S1). This suggests that its functionality may also be conservative. The *sn* gene plays an important role in insects. For example, the scales on the wings of moths and butterflies are specialized chitin layers that can sense the temperature and allow for thermoregulation [30]. Insects can develop heat stress in hot environments [31]. Heat stress produces highly conserved adaptive responses in animals [32]. Heat stress leads to the accumulation of ROS [33]. ROS accumulation causes oxidative stress, which in turn results in DNA damage, lipid oxidation, protein degradation, enzyme inactivation, and cell death [34]. In this study, we found that the *Bmsn* gene reduces heat stress-induced ROS accumulation in silkworms, reduces cellular DNA damage, improves cell viability, and prolongs cell survival. This suggests that the *Bmsn* gene is able to regulate intracellular ROS levels and increase the resistance to high temperatures in the silkworm.

The fat body is an important metabolic organ of insects, and it has a direct effect on the regulation of insect growth and development [35]. The silkworm fat body is equivalent to the adipose tissue and liver of vertebrates, and it is an important organ for nutrient storage, energy metabolism, immune response, and detoxification [36]. Our data showed that the *Bmsn* gene is highly expressed in the fat body of the silkworm (Figure 1C), and there was a significant difference in the morphology of the fat body of the *Bmsn*-OE strain and the Dazao strain after high-temperature treatment. At high temperatures, the fat body of the Dazao strain was flocculent, and the cells of fat corpuscles dissociated, while the fat body of the *Bmsn*-OE strain remained flaky, and the cells were still dense. The fat body is the center of material and energy metabolism in the silkworm. During the larval stage, the fat body absorbs a large amount of nutrients from the blood to supply the energy needed during the less active pupa and adult stages [37]. The *Bmsn* gene delays the dissociation of fat body cells at high temperatures, indicating that it participates in the response of the fat body to high-temperature stress [38].

To detail the effects of the *Bmsn* gene on individual development in silkworms exposed to high temperatures, we constructed a transgenic silkworm strain overexpressing the *Bmsn* gene. We analyzed the economic characteristics (cocoon weight and cocoon shell weight) and high-temperature resistance of the *Bmsn*-OE strain and the Dazao strain. The *Bmsn*-OE strain had a larger body size, greater weight, and higher cocoon formation rate under the same feeding conditions, which indicates that the *Bmsn* gene promotes silkworm growth and development and improves its economic characteristics. In the 27 °C group, the mortality statistics showed a small but significant difference; this result was within a reasonable range considering the influence of abiotic factors and the randomly selected growth stage of the silkworms. The high-temperature resistance of the *Bmsn*-OE strain was superior to that of the Dazao strain, with a significantly higher silkworm mortality, cocoon formation rate, and pupa life rate observed in the *Bmsn*-OE strain compared to the Dazao strain at high temperatures. It is important for the sericulture industry to improve the high-temperature resistance and economic characteristics of silkworms. Additionally, nearly half of the agricultural and forestry pests are Lepidoptera [16], suggesting that the negative effect of interfering with the *sn* gene may be significant for Lepidoptera pest control.

## 5. Conclusions

**Figure 8:**
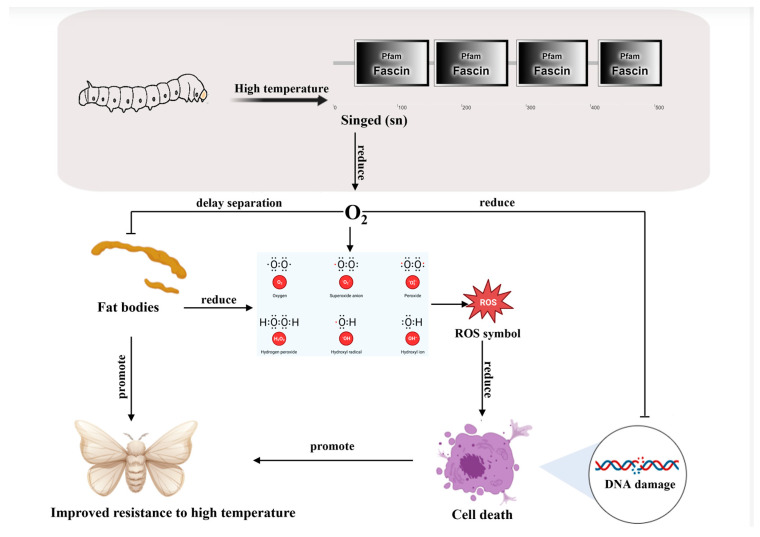
Diagram of model regulation.

To summarize, we demonstrated that the *Bmsn* gene can increase the proliferation activity of silkworm cells and enhance their tolerance to high temperatures. Overexpression of the *Bmsn* gene can increase high-temperature resistance and improve the economic characteristics of silkworms (Figure 8). The *Bmsn*-OE transgenic strain is valuable for the development of stress-resistant silkworm varieties and the advanced study of high-temperature resistance mechanisms. This study also provides new molecular targets that may be useful for the management of Lepidoptera pests.

## Figures and Tables

**Table 1:** DNA Damage Comet Assay results.

Group	Tail Length (µm + SD)	Head DNA (%)	Tail DNA (%)	Olive Tail Moments
Control (37 °C)	56.94 ± 11.49 _a_	65.98 ± 8.78 _a_	34.02 ± 8.78 _a_	24.32 ± 6.63 _a_
*Bmsn*-OE (37 °C)	34.42 ± 8.48 _bbb_	75.64 ± 7.59 _bbb_	24.36 ± 7.59 _bbb_	12.64 ± 4.58 _bbb_
Control (27 °C)	6.34 ± 5.51 _ccc_	97.96 ± 3.42 _ccc_	2.04 ± 3.42 _ccc_	1.03 ± 1.49 _ccc_
*Bmsn*-OE (27 °C)	5.26 ± 5.62 _ccc_	98.36 ± 2.65 _ccc_	1.64 ± 2.65 _ccc_	0.82 ± 1.23 _ccc_

Values that do not share a common subscript letter in each column are significantly different at the *p* < 0.001 level.

## Data Availability

Data are contained within the article and Supplementary Materials.

## Supplementary Materials

The following supporting information can be downloaded at https://www.mdpi.com/article/10.3390/insects15040264/s1. Figure S1: Prediction of Bmsn protein domain (A) and multiple sequence alignment (B); Figure S2: Semi-quantitative detection of *Bmsn* gene expression levels in tissues; Table S1: Primers used in this study.
