# Supplementary material for: The Bombyx mori singed Gene Is Involved in the High-Temperature Resistance of Silkworms
Source: Insects. 2024 Apr 12;15(4):264. doi: 10.3390/insects15040264 (PMC11049829; doi:10.3390/insects15040264)
Supplement: Supplementary file 1 [file insects-15-00264-s001.zip › Table S1.pdf]

Table S1. Primers used in this study.

Primer	Sequence
Bmsn-Flag-BamHI	F: 5' cgggatccATGGATTACAAGGATGACGACGATAAGAACGGACACAGCAATGGCGACA 3'
Bmsn-Flag-XbaI	R: 5' gctctagaCTACTTATCGTCGTCATCCTTGTAATCGTACTCCCAGTGCGTGGCGGAG 3'
pBacL	F: 5' ATCAGTGACACTTACCGCATTGACA 3'
	R: 5' TGACGAGCTTGTTGGTGAGGATTCT 3'
pBacR	F: 5' TACGCATGATTATCTTTAACGTA 3'
	R: 5' GTACTGTCATCTGATGTACCAGG 3'
Q-BmLeft Gene	F: 5' ACAAATCATTTCGCAAGGTGTC 3'
	R: 5' TTCGATTTGATCACTGGTGGTG 3'
Q-BmRight Gene	F: 5' ACACCAAGTTTCAAGTCAATCG 3'
	R: 5' AATCCCTGACTATCCTGTAACG 3'
Q-*Bmsn*	F: 5' TCCACACCTGCAACAACAAG 3'
	R: 5' GAAGAGTTCGTCCCTGGTGA 3'
Q-SW22934	F: 5' TTCGTACTGGCTCTTCTCGT 3'
	R: 5' CAAAGTTGATAGCAATTCCCT 3'
Q-*Bmsn*	F: 5' TCCACACCTGCAACAACAAG 3'
	R: 5' GAAGAGTTCGTCCCTGGTGA 3'
Q-BmSOD1	F: 5' CTTCGATCAGCAGGATGAAAAG 3'
	R: 5' CAATGATGCTGTTAGGTCCATG 3'
Q-BmSOD2	F: 5' CAAATAGCTACATGCCAGAACC 3'
	R: 5' CACGAACGTTCTTGTACTGAAG 3'
Q-BmSOD3	F: 5' AACATTATTGTCGTACGCTTG 3'
	R: 5' CATTACCAGTCGTCAGGGATAG 3'
Q-BmCAT	F: 5' TTTGCAACCTCGATACAATGTC 3'
	R: 5' TTGGCAATAGCTCGTTGTTTAG 3'
Q-BmGADD45	F: 5' GATTGTGAGACTAAGCTGAGGA 3'
	R: 5' CTGTCTCTCGGCTTCAGATAAT 3'
Q-BmGpx	F: 5' AGAGATAGTTTGCTTTGCCTCT 3'
	R: 5' GCTTCAAATACTTCCACAGTGG 3'
