# Supplementary figures and images for: The Bombyx mori singed Gene Is Involved in the High-Temperature Resistance of Silkworms
Source: Insects. 2024 Apr 12;15(4):264. doi: 10.3390/insects15040264 (PMC11049829; doi:10.3390/insects15040264)

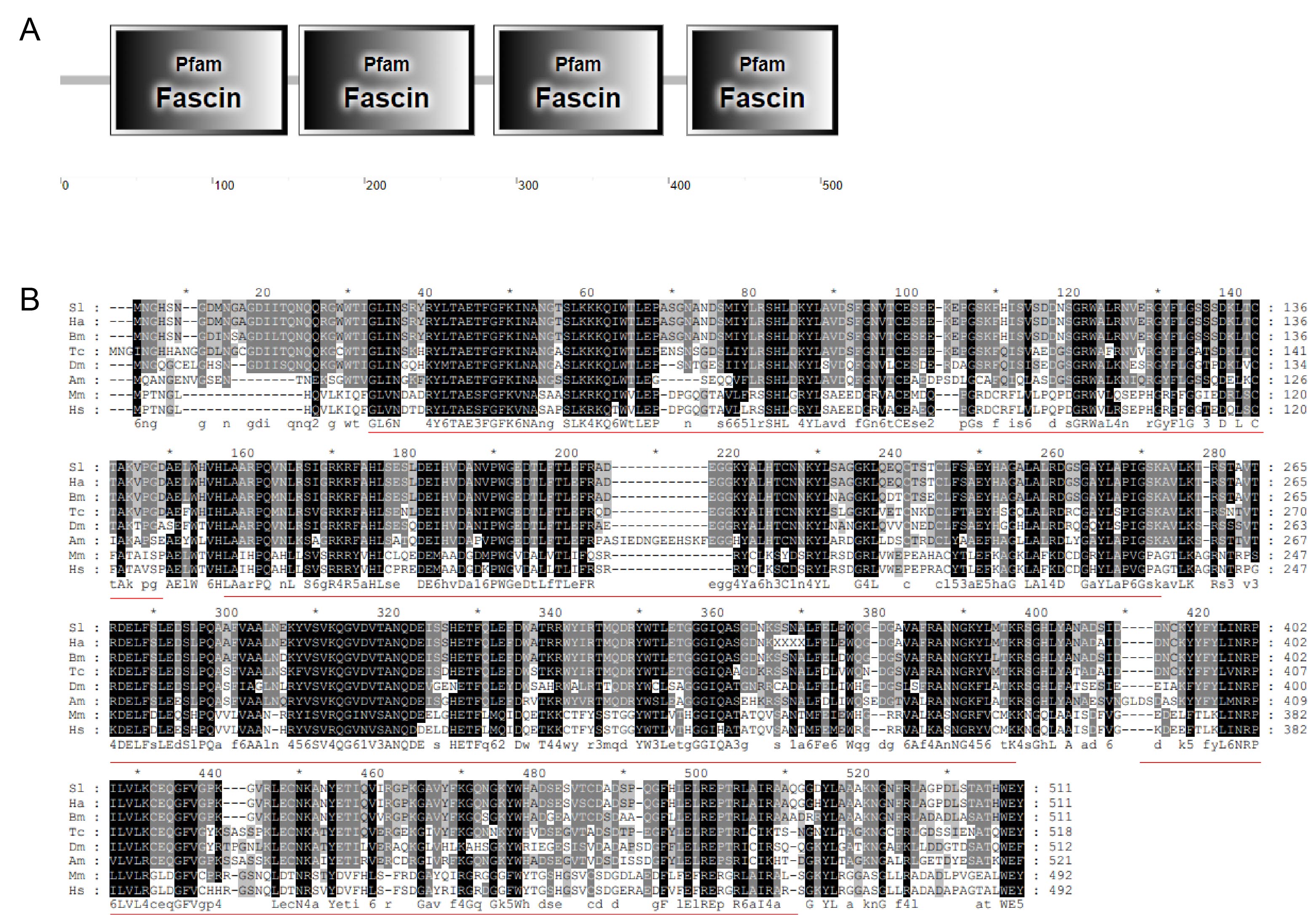

Supplement: Supplementary file 1 [file insects-15-00264-s001.zip › Figure S1.jpg]

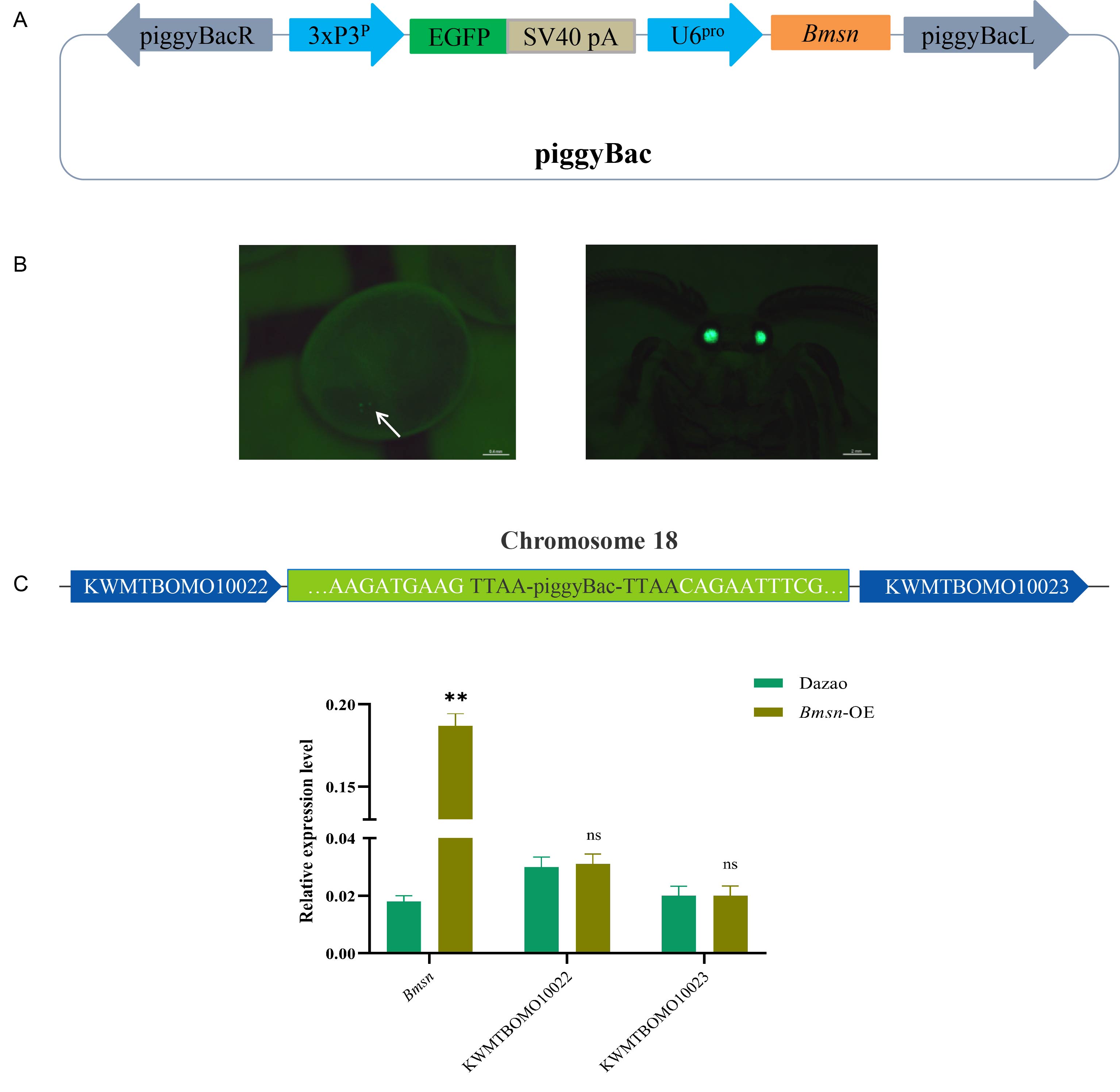

Supplement: Supplementary file 1 [file insects-15-00264-s001.zip › Figure S2.jpg]
